# Neurotoxicity mechanisms and clinical implications of six common recreational drugs

**DOI:** 10.3389/fphar.2025.1526270

**Published:** 2025-02-17

**Authors:** Jing Wang, Yulei Hao, Di Ma, Liangshu Feng, Feng Yang, Pingxu An, Xingqi Su, Jiachun Feng

**Affiliations:** ^1^ Department of Neurology and Neuroscience Center, The First Hospital of Jilin University, Changchun, Jilin, China; ^2^ Department of Neurology, Jining First People’s Hospital, Jining, Shandong, China; ^3^ Department of Oncological Neurosurgery, The First Hospital of Jilin University, Changchun, Jilin, China

**Keywords:** methamphetamine, cocaine, synthetic cathinones, ketamine, nitrous oxide, heroin, nervous system damage

## Abstract

The recreational abuse of addictive drugs poses considerable challenges to public health, leading to widespread neurotoxicity and neurological dysfunction. This review comprehensively examines the neurotoxic mechanisms, clinical manifestations, and treatment strategies associated with six commonly abused substances: methamphetamine, cocaine, synthetic cathinones, ketamine, nitrous oxide and heroin. Despite their diverse pharmacological properties, these drugs converge on shared neurotoxic pathways, including oxidative stress, mitochondrial dysfunction, excitotoxicity, and neuroinflammation. Psychostimulants, such as methamphetamine, cocaine and synthetic cathinones, disrupt monoaminergic neurotransmission, causing cognitive impairment, psychiatric disturbances, and neurovascular damage. Dissociative anesthetics, including ketamine and nitrous oxide, impair glutamatergic transmission and mitochondrial function, thereby exacerbating excitotoxicity and neuronal apoptosis. Opioids, such as heroin, primarily target the brain’s reward system and induce oxidative stress, neuroinflammation, and cerebrovascular complications. Treatment strategies remain limited, focusing on symptomatic management, neuroprotective interventions, and behavioral therapies. Emerging approaches, such as antioxidants, NMDA receptor modulators, and cognitive rehabilitation, show promise but require further validation. By highlighting the underlying mechanisms and therapeutic challenges, this review provides a foundation for developing targeted interventions and advancing research on drug-induced neurotoxicity.

## 1 Introduction

The global increase in recreational drug use continues to pose significant challenges to public health and societal wellbeing. A 2022 report by the United Nations Office on Drugs and Crime documented a sharp increase in global drug use, with 292 million individuals reporting the recreational use of substances, representing a 20% increase over the past decade. These substances not only induce physical dependence and psychological addiction but also contribute to severe neurotoxicity, manifesting as persistent cognitive impairment, emotional instability, and motor dysfunction.

This review focuses on six commonly abused drugs (Table 1), classified based on their pharmacological and neurotoxic profiles: Psychostimulants: Methamphetamine (METH), cocaine and synthetic cathinones (“bath salts”) primarily act on monoaminergic systems, leading to disruptions in dopamine, serotonin, and norepinephrine signaling (Sulzer et al., 1995; Hadlock et al., 2011). These substances induce neurotoxicity through oxidative stress, excitotoxicity, and neuroinflammation, often resulting in cognitive and psychiatric impairments. Dissociative anesthetics: Ketamine and N_2_O predominantly affect glutamatergic transmission by antagonizing NMDA receptors (NMDARs), causing excitotoxicity, mitochondrial dysfunction, and neuronal apoptosis. Opioids: Heroin (Obara et al., 2013), as a potent μ-opioid receptor agonist, disrupts reward pathways and contributes to neuroinflammation and oxidative stress.

**TABLE 1:** The recreational abuse of six addictive drugs: mechanisms, clinical manifestations, and treatment.

Drug	Category	Mechanism of Action	Neurotoxic Manifestations	Key Molecular Pathways/Mechanisms	Therapeutic Strategies	References
METH	Psychostimulant	Increases dopamine release and inhibits dopaminere uptake	Cognitive impairment, psychiatric symptoms, motor dysfunction	Oxidative stress, mitochondrial dysfunction, excitotoxicity, neuroinflammation, autophagy and apoptosis	CBT, atomoxetine, modafinil, immunotherapy	Sulzer et al. (1995) Davidson et al. (2001)
Cocaine	Psychostimulant	Blocks monoamine neurotransmitter reuptake (dopamine, norepinephrine, serotonin)	Vascular damage, stroke, cognitive deficits, psychiatric symptoms	Mitochondrial dysfunction, neuroinflammation excitotoxicity	Calcium hannel blockers, M-CBT, anti-epileptic treatment	Liu et al. (2022) Malcolm et al. (2002)
Synthetic Cathinones	Psychostimulant	Enhances monoamine neurotransmitter release and inhibits reuptake	Behavioral disorders, anxiety, cognitive impairment	Oxidative stress, mitochondrial dysfunction, excitotoxicity	-	Simmler et al. (2013) Leong et al. (2020)
Ketamine	Dissociativeanesthetic	Non-competitive NMDA receptor antagonist disrupting glutamatergic transmission	Cognitive deficits, neuroinflammation, motor dysfunction	Neuroinflammation, mitochondrial dysfunction, glutamate excitotoxicity	miR-34a modulation, 17β-estradiol supplementation, L-carnitine administration	Wan et al. (2024) Li et al. (2013)
Nitrous Oxide (N₂O)	Dissociativeanesthetic	Inhibits NMDA receptors and inactivates Vitamin B12	Spinal cord lesions, peripheral neuropathy cognitive deficits	Oxidative stress, excitotoxicity, methylation dysfunction demyelination	Vitamin B12 supplementation folic acid herapy	Purger et al. (2016) Marotta and Kesserwani (2020)
Heroin	Opioid	Activates μ-opioid receptors, modulating dopamine release	Cognitive deficits, stroke, leukoencephalopathy	Oxidative stress, neuroinflammation, mitochondrial dysfunction	Calcium hannel blockers, naltrexone	Nobay et al. (2004) Peyravian et al. (2022)

Each of these substances operates through distinct molecular and cellular mechanisms but shares common pathways of neurotoxicity. For example, METH and cocaine disrupt monoaminergic signaling, whereas ketamine and nitrous oxide impair glutamatergic neurotransmission. Similarly, synthetic cathinones mimic the effects of traditional stimulants but exhibit heightened potency and toxicity.

Despite the increasing recognition of the neurological damage caused by these substances, effective treatment strategies remain limited. Current approaches, including pharmacological and behavioral interventions, often focus on mitigating symptoms rather than addressing the underlying mechanisms of damage. By systematically reviewing the neurotoxic mechanisms, clinical manifestations, and therapeutic interventions associated with these six drugs, this study aims to provide insights into the current knowledge gaps and potential avenues for future research.

## 2 METH

METH, widely known as “crystal meth,” is a potent psychostimulant and one of the most abused illicit drugs globally. According to a 2022 United Nations Office on Drugs and Crime report, METH use has markedly increased, particularly in North America, East Asia, and Oceania, where it accounts for a significant proportion of stimulant-related drug abuse cases. In 2020 alone, an estimated 34 million individuals worldwide engaged in amphetamine-type stimulant use, highlighting the growing burden of METH abuse on public health systems.

METH is predominantly abused in its hydrochloride salt form, which appears as a colorless crystalline substance that is highly soluble in water and ethanol. Its free-base form, although less common, exhibits increased volatility and solubility in organic solvents.

Chronic METH abuse causes severe and persistent damage to the central nervous system (CNS), leading to cognitive deficits, psychiatric symptoms, and motor dysfunction. Understanding the mechanisms underlying METH-induced neurotoxicity is essential for developing effective treatment strategies to mitigate its devastating effects.

### 2.1 Pharmacological effects

METH is a lipophilic compound that rapidly crosses the blood-brain barrier (BBB) and accumulates in the brain. Its pharmacological effects stem primarily from the disruption of monoaminergic neurotransmission and its downstream neurotoxic consequences.

#### 2.1.1 Monoamine transporter disruption and neurotransmitter release

METH disrupts the reuptake and enhances the release of dopamine, serotonin, and norepinephrine, significantly increasing their extracellular concentrations. Furthermore, methamphetamine blocks monoamine transporters and inhibits monoamine oxidase activity, crucial for the breakdown of neurotransmitters within the neurons. It also induces the reverse transport of these transporters (Sulzer et al., 1995), leading to the release of monoamines from the neuronal cytoplasm into the synaptic cleft (McCann et al., 1998). This results in excessive release of dopamine, norepinephrine, and serotonin, which overstimulate postsynaptic receptors and produce the euphoric and stimulant effects associated with METH use (Lee et al., 2009; Okita et al., 2018).

#### 2.1.2 Vesicular monoamine transporter 2 inhibition

METH inhibits vesicular monoamine transporter 2 and disrupts monoamine storage in synaptic vesicles (Eyerman and Yamamoto, 2007; Lizarraga et al., 2015). The resulting accumulation of cytoplasmic dopamine promotes dopamine autoxidation, generating reactive oxygen species (ROS) and dopamine quinones that contribute to oxidative stress.

#### 2.1.3 Calcium dysregulation and excitatory neurotransmission

METH enhances glutamate release, leading to NMDAR overactivation and increased calcium influx into neurons (Staszewski and Yamamoto, 2006). Elevated calcium levels activate calpains and other calcium-dependent enzymes, which impair mitochondrial function and contribute to neuronal injury.

#### 2.1.4 BBB disruption

METH compromises BBB integrity, allowing peripheral immune cells to infiltrate the CNS (Ramirez et al., 2009). This sets the stage for neuroinflammation, which amplifies oxidative stress and excitotoxicity, as discussed in subsequent sections.

### 2.2 Neurotoxic mechanisms

METH induces neurotoxicity via a combination of oxidative stress, mitochondrial dysfunction, excitotoxicity, and neuroinflammation. These processes are closely interrelated, amplifying each other and contributing to long-term neuronal damage and neurodegeneration.

#### 2.2.1 Oxidative stress and mitochondrial dysfunction

**FIGURE 1:**
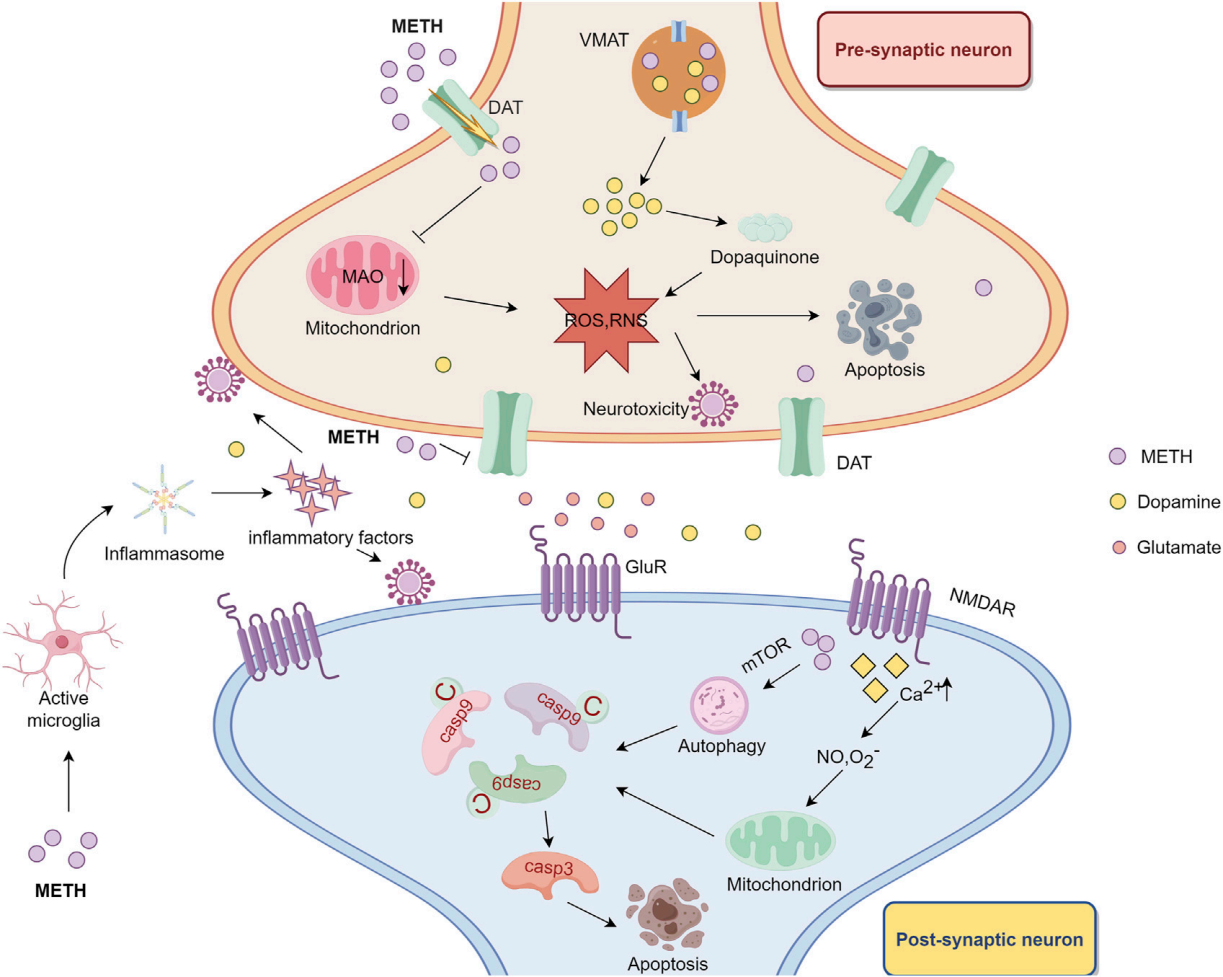
The neurotoxic mechanism of methamphetamine. Methamphetamine induces oxidative stress by increasing the production of ROS and reducing the activity of antioxidant enzymes, thereby impairing mitochondrial function, causing a rise in mitochondrial ROS production and contributing to cellular dysfunction and apoptosis. Additionally, methamphetamine’s toxicity involves the autoxidation of dopamine, producing dopamine quinone and further ROS, which damages axon terminals and leads to neuronal terminal damage. This toxicity is exacerbated by increased excessive production of glutamate and calcium. By Figdraw.

METH significantly increases the production of ROS and reactive nitrogen species, overwhelming antioxidant defenses in neurons. This oxidative stress is particularly damaging because of METH’s effects on dopamine, which undergoes autoxidation, leading to the generation of dopamine quinones and ROS (Davidson et al., 2001). These byproducts damage cellular structures, including proteins, lipids, and DNA, and contribute to neuronal dysfunction. Oxidative stress disrupts mitochondrial function by impairing the electron transport chain and inducing mitochondrial permeability transition (Berman and Hastings, 1999). Mitochondrial dysfunction increases ROS production and creates a vicious cycle that exacerbates neuronal injury (Brown et al., 2005). As mitochondria fail to maintain cellular energy, neuronal survival is compromised, and apoptotic pathways are activated (Davidson et al., 2001) (Figure 1).

#### 2.2.2 Excitotoxicity and neuroinflammation

Excitotoxicity is another major contributor to METH-induced neurotoxicity and is driven by an imbalance in glutamate neurotransmission. METH stimulates the release of excess glutamate from neurons while inhibiting its uptake by astrocytes, leading to the overactivation of NMDARs (Northrop et al., 2011). This overactivation causes an influx of calcium ions into neurons, which, in turn, activates calcium-dependent enzymes, such as calpain, nitric oxide synthase and phosphatase, resulting in the degradation of cytoskeletal proteins and damage to cell membranes. Activated nitric oxide synthase facilitates the production of nitric oxide, which reacts with hydrogen peroxide to yield the dangerous radical peroxynitrite. Peroxynitrite has been identified as one of the main executors of cellular damage. The elevated calcium levels also impair mitochondrial function, triggering the release of proapoptotic factors, including cytochrome c, and activating caspase-dependent apoptotic pathways (Jayanthi et al., 2004). The combination of excitotoxicity and mitochondrial dysfunction accelerates neuronal death.

tThe excitotoxicity induced by METH also triggers neuroinflammation and amplifies its neurotoxic effects. The overstimulation of neurons activates microglia, the resident immune cells of the brain, which release proinflammatory cytokines, such as TNF-α and IL-1β, as well as ROS (McConnell et al., 2015) (Figure 1). These inflammatory mediators further damage neurons and disrupt the BBB, allowing peripheral immune cells to infiltrate the CNS (Ramirez et al., 2009). Additionally, astrocytes, which are normally involved in clearing excess glutamate and supporting neuronal function, become reactive in response to toxic environments. Reactive astrocytes exacerbate inflammation by releasing additional cytokines, contributing to a feedback loop that worsens neuronal injury (Du et al., 2017).

The interaction between excitotoxicity and neuroinflammation is critical for the development of long-term neurodegeneration. Persistent activation of both microglia and astrocytes creates an environment that supports ongoing neuronal damage, leading to chronic neuroinflammation and cognitive deficits.

#### 2.2.3 Autophagy and apoptosis

Autophagy is an early cellular response to METH-induced stress and is activated by the mTOR signaling pathway (Kongsuphol et al., 2009). Although autophagy can help remove damaged organelles and proteins, prolonged activation in response to METH leads to the degradation of essential cellular components, contributing to neuronal death (Xu et al., 2018) (Figure 1). Additionally, the combined effects of oxidative stress and excitotoxicity activate both caspase-dependent and caspase-independent apoptotic pathways, leading to cellular fragmentation and neurodegeneration. Recent studies have highlighted METH-induced alterations in the hippocampus, such as reduced STX17 expression, impaired autophagosome maturation, and cognitive decline (Wang et al., 2024).

### 2.3 Manifestations of nervous system injury

METH abuse induces neurological damage that manifests as cognitive impairment, psychiatric symptoms, and motor dysfunction, with clear distinctions between the acute and chronic phases of use. In the acute phase, symptoms include euphoria, anxiety, paranoia, and psychomotor agitation, which are primarily caused by the short-term overactivation of neurotransmitter systems (Cruickshank and Dyer, 2009). By contrast, the chronic phase is characterized by persistent cognitive decline, worsening psychiatric symptoms, and progressive motor dysfunction as neuroinflammation and neuronal loss become more pronounced (McCann et al., 2008).

#### 2.3.1 Cognitive impairment

METH abuse significantly impairs cognitive function by targeting key brain regions (McCann et al., 2008), such as the hippocampus, striatum, amygdala, and prefrontal cortex. In the acute phase, METH causes an excessive release of dopamine and glutamate, resulting in transient deficits in attention and working memory (Mark et al., 2004). However, chronic exposure leads to irreversible neuronal loss and synaptic damage, manifesting as persistent memory, executive functioning, and decision-making deficits (North et al., 2013). Neuroimaging studies have shown a reduction in hippocampal volume and abnormal prefrontal cortical activity, which strongly correlates with impairments in verbal memory and information processing speed (Chang et al., 2007).

#### 2.3.2 Psychiatric symptoms

METH abuse disrupts the functionality of various brain regions, including the prefrontal cortex, nucleus accumbens, and thalamus, leading to significant psychiatric disturbances (Sekine et al., 2001). During the acute phase, the METH-induced overactivation of the dopaminergic system causes euphoria, anxiety, and paranoid ideation (Cruickshank and Dyer, 2009). With chronic abuse, these effects progress into schizophrenia-like symptoms, such as hallucinations, delusions, and psychosis, which are exacerbated by prolonged neurotoxic effects (Hsieh et al., 2014). Chronic METH exposure often results in depression, mood instability, and impulse control disorders, reflecting long-term disruptions in neurotransmitter balance and neuroinflammatory responses (Moallem et al., 2018).

#### 2.3.3 Motor dysfunction

Motor dysfunction caused by METH abuse is commonly characterized by parkinsonian-like symptoms, including tremors, bradykinesia, and rigidity (Moallem et al., 2018). During the acute phase, elevated dopamine levels disrupt motor coordination, resulting in psychomotor agitation and involuntary movements. Chronic exposure exacerbates these symptoms through mechanisms, such as oxidative stress, excitotoxicity, and mitochondrial dysfunction, in the nigrostriatal pathway (Graves et al., 2021), leading to progressive motor slowing and postural instability. Furthermore, experimental studies have shown that METH decreases the expression of myelin basic protein (MBP) and CDK5, contributing to demyelination (Yang et al., 2018). This process may cause symptoms, such as numbness, tingling, and limb weakness, although clinical confirmation remains limited.

### 2.4 Auxiliary examinations

Auxiliary examinations, particularly neuroimaging and neurochemical analyses, play a crucial role in identifying functional and structural changes in the nervous system caused by METH abuse. These findings provide objective evidence supporting the previously discussed clinical manifestations and underlying neurotoxic mechanisms.

Neuroimaging studies, including positron emission tomography (PET) and magnetic resonance imaging (MRI), have consistently demonstrated widespread abnormalities in brain regions involved in cognitive control, emotional regulation, and motor function. PET scans have revealed a significant reduction in dopamine levels in the striatum, including the caudate nucleus and putamen, which disrupted dopamine signaling. This reduction correlated with impaired motor function and the development of compulsive drug-seeking behaviors (Sekine et al., 2001). Concurrently, altered glucose metabolism in the orbitofrontal cortex and limbic regions reflects energy deficits and mitochondrial dysfunction, contributing to the severity of psychiatric symptoms, such as paranoia, agitation, and mood disturbances (Chang et al., 2007).

MRI studies have further identified gray matter atrophy in the prefrontal cortex, medial cingulate gyrus, and hippocampus, regions that are critically involved in memory, decision-making, and emotional regulation (Thompson et al., 2004). In particular, hippocampal damage is correlated with memory impairment and learning difficulties, whereas prefrontal atrophy contributes to deficits in executive function and increased impulsivity. In addition, diffusion tensor imaging highlighted reduced white matter integrity within the corpus callosum and thalamic radiation, suggesting demyelination and impaired neural connectivity. These changes can result in slower information processing, reduced motor coordination, and increased cognitive inflexibility (Yang et al., 2018).

Magnetic resonance spectroscopy provides complementary biochemical evidence of METH-induced neurotoxicity. Reduced concentrations of N-acetyl aspartate and total creatine in the basal ganglia are markers of neuronal loss and impaired energy metabolism, which correlate with cognitive dysfunction and motor deficits (Nordahl et al., 2005). Conversely, increased levels of choline and myo-inositol in the prefrontal cortex suggest neuroinflammation and glial activation, consistent with the inflammatory processes observed in METH-induced neurotoxicity (Chang et al., 2007).

Behavioral assessments aligned closely with these imaging findings, offering a clinical perspective on the neurological damage caused by METH abuse. Chronic METH users exhibit significant deficits in working memory, executive function, and attention, which reflect structural and functional changes in the prefrontal cortex and hippocampus (McCann et al., 2008). Motor impairments, including fine motor dysfunction, reduced coordination, and slower reaction times, are consistent with basal ganglia damage and white matter disruption. Psychiatric symptoms, such as paranoia, anxiety, and psychosis, further correlate with the observed abnormalities in the limbic and orbitofrontal regions (Zweben et al., 2004).

Collectively, these neuroimaging and behavioral findings illustrate the extensive impact of METH on both brain structure and function. They provide a clear link between biochemical disruptions, such as dopamine depletion and neuroinflammation, and the resulting cognitive, psychiatric, and motor symptoms observed in chronic METH abusers.

### 2.5 Treatment

Cognitive-behavioral therapy (CBT) is a well-established standard treatment for addressing cognitive impairment caused by METH abuse (Lee and Rawson, 2008). CBT primarily improves cognitive deficits through structured psychosocial interventions targeting behavioral patterns and decision-making processes. Although CBT has shown considerable promise, its long-term effectiveness can be influenced by factors, such as treatment adherence, patient motivation, and the availability of resources for prolonged interventions (Apuy et al., 2023).

In addition to behavioral therapies, pharmacological approaches have demonstrated potential benefits. A double-blind randomized controlled trial revealed that atomoxetine, a selective norepinephrine reuptake inhibitor, effectively enhanced cognitive functions, such as memory, inhibitory control, and attention, in METH users (Rabiey et al., 2022). Similarly, modafinil, a dopamine reuptake inhibitor, has been shown to improve recognition memory impairment in METH users. However, the beneficial effects of modafinil have been primarily observed in mice, and further clinical studies are necessary to validate its efficacy and safety in humans (González et al., 2014).

Immunotherapy strategies, such as MHC/neuroantigen peptide constructs, have emerged as experimental approaches for treating METH-induced learning and memory deficits. Although preclinical data show promise (Loftis et al., 2013), their clinical applicability remains unclear owing to challenges, such as immune response variability and limited human trials. Similarly, the neuroprotective agent baicalin has demonstrated potential in mitigating METH-induced amnesia and cognitive impairment in animal studies; however, these findings were primarily derived from experiments in rodent models, which limits their immediate translational impact (Wong et al., 2014).

In addressing motor dysfunction induced by METH, transcranial magnetic stimulation has shown potential for restoring motor control by modulating cortical excitability (Edinoff et al., 2023). Although transcranial magnetic stimulation is a noninvasive and promising intervention, its accessibility, treatment duration, and cost-effectiveness remain significant concerns for real-world clinical applications. In preclinical studies, the fragment C domain of the tetanus toxin (Hc-TeTx) has been shown to alleviate METH-induced neurotoxicity and motor deficits in animal models; however, human studies are lacking thus far (Mendieta et al., 2016).

Additionally, physical exercise has been recognized as an effective adjunctive intervention for mitigating the cognitive and motor impairments associated with chronic METH use. Exercise modulates dopamine levels and enhances neuroplasticity, offering a nonpharmacological approach for long-term recovery (Morais et al., 2018). However, its efficacy may vary depending on patient compliance, exercise intensity, and duration, which poses challenges to its widespread implementation.

While these treatment approaches, both behavioral and pharmacological, hold promise for alleviating METH-induced neurological damage, their long-term efficacy, potential side effects, and real-world feasibility remain significant concerns. Future clinical trials and translational studies are required to better evaluate these interventions and establish comprehensive treatment protocols for METH users.

## 3 Cocaine

Cocaine, a powerful psychostimulant derived from the coca plant, dissolves in water and ethanol but not in typical organic solvents, such as ether. As a psychostimulant, it exerts its effects primarily by influencing monoamine neurotransmitters (Xu et al., 2013). Cocaine neurotoxicity and neurological damage stem from several mechanisms, including mitochondrial dysfunction (Cunha-Oliveira et al., 2010), oxidative stress (Poon et al., 2007), neuroinflammation (Correia et al., 2020), excitotoxicity (Moran et al., 2005), and autophagy (Cao et al., 2017).

### 3.1 Vascular effects of cocaine

Cocaine impairs vascular function by causing vasoconstriction, reducing cerebral blood flow, and decreasing tissue oxygenation. These effects are partly due to the prolonged activation of astrocytes (Liu et al., 2022). Animal studies have shown that cocaine use leads to cortical microischemia and prefrontal cortical ischemia (Ren et al., 2012), which disrupt the neurovascular network and impair brain function (Du et al., 2020). Chronic use may also trigger cerebral angiogenesis via the HIF-VEGF pathway as a compensatory response to ischemia (Yin et al., 2017). Although angiogenesis may initially appear beneficial by enhancing neuroplasticity, it can also exacerbate vascular pathology by destabilizing the BBB.

Additionally, cocaine induces apoptosis in cerebral vascular smooth muscle cells, contributing to vasoconstriction and ischemia, resulting in significant neurological damage (Fonseca and Ferro, 2013). Cocaine-induced vasoconstriction and vasculitis can lead to severe neurological complications, including headaches, seizures, aneurysms, strokes, and subarachnoid hemorrhages (Farooque et al., 2020).

### 3.2 Neurological damage and psychiatric symptoms

The neurological effects of cocaine include diffuse leukoencephalopathy, which is characterized by vasospasms, cerebral ischemia, and posterior reversible leukoencephalopathy (Rowbotham and Lowenstein, 1990; Li et al., 2018). It also induces psychiatric symptoms, such as euphoria, hypervigilance, interpersonal sensitivity, impaired judgment, paranoid delusions, hallucinations, and bizarre behaviors (Roncero et al., 2013). Chronic cocaine exposure has been linked to an increased risk of Parkinson’s disease, as it promotes α-synuclein overexpression in dopamine neurons, causing resting tremors and neurotoxic damage (Mash et al., 2003).

Imaging studies have demonstrated that chronic cocaine users often exhibit brain atrophy and reduced gray and white matter volumes, particularly in the limbic regions essential for emotional processing and reward (Bartzokis et al., 2000; Rando et al., 2013).

### 3.3 Treatment strategies

Calcium channel blockers, such as amlodipine, have shown promise in preventing cocaine-induced vascular toxicity by lowering blood pressure and reducing headache frequency (Malcolm et al., 2002; Du et al., 2021). Decompressive craniectomy is an effective treatment for cocaine-induced ischemic stroke (Algahtani et al., 2021). Modified CBT addresses the cognitive dysfunction associated with cocaine abuse (Aharonovich et al., 2018). In emergencies involving agitation, rapid symptom control can be achieved using lorazepam or midazolam injections (Nobay et al., 2004). Seizures, if recurrent, should be treated with intravenous benzodiazepines, and status epilepticus should be managed according to established protocols (Meierkord et al., 2010).

## 4 Synthetic cathinones

Synthetic cathinones, colloquially known as “bath salts,” exhibit profound neurotoxic effects by affecting monoaminergic neurotransmission, oxidative stress, excitotoxicity, and neuroinflammation. These mechanisms not only disrupt neuronal integrity but also contribute to persistent cognitive and behavioral deficits.

### 4.1 Disruption of monoaminergic neurotransmission

Synthetic cathinones primarily enhance the release and inhibit the reuptake of monoamines, such as dopamine, norepinephrine, and serotonin. This results in excessive synaptic monoamine concentrations, the overstimulation of postsynaptic receptors, and excitatory neurotoxicity. For instance, mephedrone increases both dopamine and serotonin release, whereas α-PVP primarily targets dopamine and norepinephrine transporters (Simmler et al., 2013). Chronic exposure to these drugs depletes presynaptic monoamine stores, impairs long-term neuronal signaling, and induces neurodegeneration (Angoa-Perez and Kuhn, 2024).

### 4.2 Oxidative stress and mitochondrial dysfunction

The excessive release of monoamines promotes ROS formation through the auto-oxidation of dopamine and norepinephrine. This overwhelms the neuronal antioxidant defenses, causing oxidative damage to lipids, proteins, and DNA. Synthetic cathinones also impair mitochondrial function, reduce ATP production, and increase the opening of mitochondrial permeability transition pores. This cascade leads to neuronal apoptosis and energy deficits, particularly in the prefrontal cortex and hippocampus (Leong et al., 2020).

### 4.3 Excitotoxicity

Synthetic cathinones disrupt glutamatergic signaling by enhancing glutamate release and impairing astrocytic uptake (Leong et al., 2020). Elevated extracellular glutamate levels activate NMDA and AMPA receptors, resulting in excessive calcium influx into neurons (Hu et al., 2004). Calcium overload triggers the activation of calcium-dependent enzymes, including calpains and caspases, which degrade cytoskeletal proteins and lead to neuronal death (Zhang and Bhavnani, 2006).

### 4.4 Neuroinflammation

The neurotoxic environment created by synthetic cathinones activates microglia and astrocytes, leading to the release of proinflammatory cytokines, such as IL-1β and TNF-α. This inflammatory response exacerbates the neuronal damage and promotes chronic neuroinflammation (Marusich et al., 2022). Studies have shown that α-PVP and mephedrone significantly increase microglial activation in the striatum, contributing to long-term neurodegeneration (Buzhdygan et al., 2021).

## 5 Ketamine

Ketamine, first synthesized in the 1960s as a safer alternative to phencyclidine for anesthesia, has gained widespread use as both a therapeutic agent and recreational drug. Ketamine was initially introduced as an anesthetic and analgesic for medical applications (Domino, 2010). Recently, its potential for treating treatment-resistant depression has been recognized (Hultman et al., 2018), adding another layer to its therapeutic profile. However, ketamine’s hallucinogenic and dissociative effects have also led to significant recreational abuse, commonly referred to as “K powder.” This dual identity underscores the importance of examining pharmacological mechanisms, neurotoxic risks, and clinical implications under various conditions of use.

### 5.1 Pharmacological effects

#### 5.1.1 NMDAR antagonism

Ketamine exerts its primary effects by acting as a noncompetitive antagonist of NMDARs and reducing glutamate-mediated excitatory neurotransmission (Franks and Lieb, 1994; Zhou and Tajima, 2023). This mechanism underlies its anesthetic, analgesic, and antidepressant properties. However, prolonged or high-dose ketamine exposure induces a compensatory upregulation of NMDARs, increasing intracellular calcium ion influx and ROS production, ultimately leading to neuronal apoptosis (Liu et al., 2013).

In animal studies, antagonism of NMDA receptors in intermediate neurons of the forebrain and thalamus reduces GABAergic function. This action promotes the firing of layer V pyramidal neurons and stimulates glutamate release (Aleksandrova and Phillips, 2021), leading to excessive extracellular glutamate. This cascade can exacerbate neurodegeneration, particularly after chronic or high-dose administration (Liu et al., 2013). Importantly, these mechanisms have primarily been demonstrated in animal models, and their direct applicability to human pathology remains an area of active research.

#### 5.1.2 Impact on neurotransmitters and synaptic plasticity

Chronic ketamine use disrupts the balance between excitatory and inhibitory neurotransmission. For instance, in preclinical models, long-term ketamine exposure increases the expression of GABA receptor subunits in the prefrontal cortex while simultaneously reducing glutamate receptor subunits, impairing synaptic signaling and cognitive function (Luo et al., 2021). Furthermore, ketamine alters key signaling pathways, such as the CaMKIIβ-ERK1/2-CREB/NF-κB axis, leading to reduced synaptic protein expression, synaptic plasticity impairment, and neurodegeneration (Luo et al., 2021).

Ketamine also downregulates brain-derived neurotrophic factor, particularly in the hippocampus, exacerbating deficits in synaptic plasticity and cognitive functions. These neurotoxic effects may persist even after the cessation of drug use, as shown in rodent studies, highlighting the long-term impact of ketamine on brain function (Wan et al., 2024).

### 5.2 Neurotoxic mechanisms

#### 5.2.1 Neuroinflammation

Ketamine-induced neurotoxicity is associated with neuroinflammation. Chronic ketamine exposure in rodent models (50 mg/kg daily for 8 weeks) leads to significant activation of microglial cells and elevated expression of proinflammatory cytokines, such as IL-6 and IL-1β, which correlate with hippocampal neuronal damage (Yin et al., 2022). Additionally, ketamine activates the NLRP3 inflammasome, inducing caspase-1 activation and neuronal pyroptosis, further contributing to neuroinflammation in regions, such as the hippocampus and prefrontal cortex (Zhang et al., 2021).

In human studies, long-term recreational ketamine use has been associated with structural brain changes, including reduced prefrontal gray matter volume and impaired white matter connectivity, which are potentially linked to chronic low-grade inflammation (Liao et al., 2011; Edward Roberts et al., 2014).

#### 5.2.2 Mitochondrial dysfunction

Ketamine disrupts mitochondrial function by reducing the membrane potential, increasing cytochrome c release, and promoting ROS generation, which collectively induce neuronal apoptosis (Bai et al., 2013). In animal models, high-dose ketamine (100 mg/kg daily) caused mitochondrial swelling, DNA damage, and ATP production deficits (Huang et al., 2024). These mitochondrial impairments are closely associated with the disruption of energy metabolism and neuronal death.

Human studies have identified metabolic abnormalities in ketamine abusers, particularly in the prefrontal cortex, which may reflect mitochondrial dysfunction (Liao et al., 2011). Symptoms, such as chronic fatigue and memory impairment, among recreational users further underscore the clinical relevance of mitochondrial damage (Edward Roberts et al., 2014).

#### 5.2.3 Dose and frequency dependence

The neurotoxic effects of ketamine are highly dependent on its dosage and frequency of use. Recreational users often consume high doses (>2 mg/kg) with frequent administration (daily or multiple times per day), leading to pronounced neurotoxic effects, such as gray matter atrophy and white matter disintegration (Liao et al., 2011). By contrast, low doses (0.5 mg/kg) administered intermittently for therapeutic purposes are considered relatively safe in the short term but may still pose risks with repeated use (Smith-Apeldoorn et al., 2022).

#### 5.2.4 Age, genetics, and comorbidities

Individual susceptibility to the neurotoxic effects of ketamine varies according to age, genetic factors, and comorbidities. Adolescents whose brains are still developing are more vulnerable than adults to ketamine-induced neuronal apoptosis and cognitive deficits (Yin et al., 2022). Genetic polymorphisms in NMDAR subunits, such as NR1 and NR2B, influence individual sensitivity to the therapeutic and toxic effects of ketamine (Smith-Apeldoorn et al., 2022).

Comorbid conditions, such as diabetes and chronic kidney disease, amplify ketamine’s neurotoxicity by increasing oxidative stress and impairing drug metabolism. These factors contribute to mitochondrial dysfunction and cognitive impairment in affected individuals (Edward Roberts et al., 2014).

### 5.3 Manifestations of neuropsychiatric system injury

Ketamine abuse commonly manifests as dissociative symptoms, such as altered consciousness, detachment from the environment, and sensory loss (Vlisides et al., 2018). High doses may lead to the “K-hole” phenomenon, characterized by extreme dissociation without loss of consciousness (Malhotra et al., 1996). Chronic abuse is associated with cognitive impairments, including memory and attention deficits, psychotic-like symptoms (hallucinations and delusions), and motor dysfunction (Morgan et al., 2004).

### 5.4 Auxiliary examination

Neuroimaging studies have provided critical insights into ketamine-induced neurotoxicity. PET scans have revealed altered cerebral blood flow, with increases in regions, such as the anterior cingulate cortex, and decreases in the cerebellum (Holcomb et al., 2001). MRI studies have demonstrated reduced prefrontal gray matter volume and impaired white matter integrity in chronic users (Liao et al., 2011). In severe cases, CT scans reveal widespread brain atrophy (Liu et al., 2021).

### 5.5 Treatment

Gradual tapering of ketamine is recommended to minimize withdrawal symptoms because abrupt cessation may result in hyperalgesia and sensory disturbances (Mitchell, 1999). Experimental approaches, such as miR-34a modulation, 17β-estradiol supplementation, and L-carnitine administration, show promise in mitigating ketamine-induced neurotoxicity (Li et al., 2013; Liu et al., 2013). Although most cognitive impairments resolve after cessation, episodic memory and attention deficits may persist, highlighting the need for long-term follow-up (Morgan et al., 2004).

## 6 N₂O

N₂O, commonly referred to as laughing gas, is widely used in both medical and industrial settings, such as anesthesia and food processing. Despite its utility, N₂O abuse has increased because of its ability to induce brief euphoria when inhaled. N₂O is a colorless, liquefiable gas with a slightly sweet odor and taste. Although chemically stable, it has a significant impact on neurological and biochemical processes, particularly through its interaction with vitamin B12 (Vit B12).

To better understand the multifaceted impact of N_2_O, this article examines its molecular biological mechanisms, neural damage pathways, and clinical manifestations, highlighting the interconnections between these aspects.

### 6.1 Molecular biology mechanism

**FIGURE 2:**
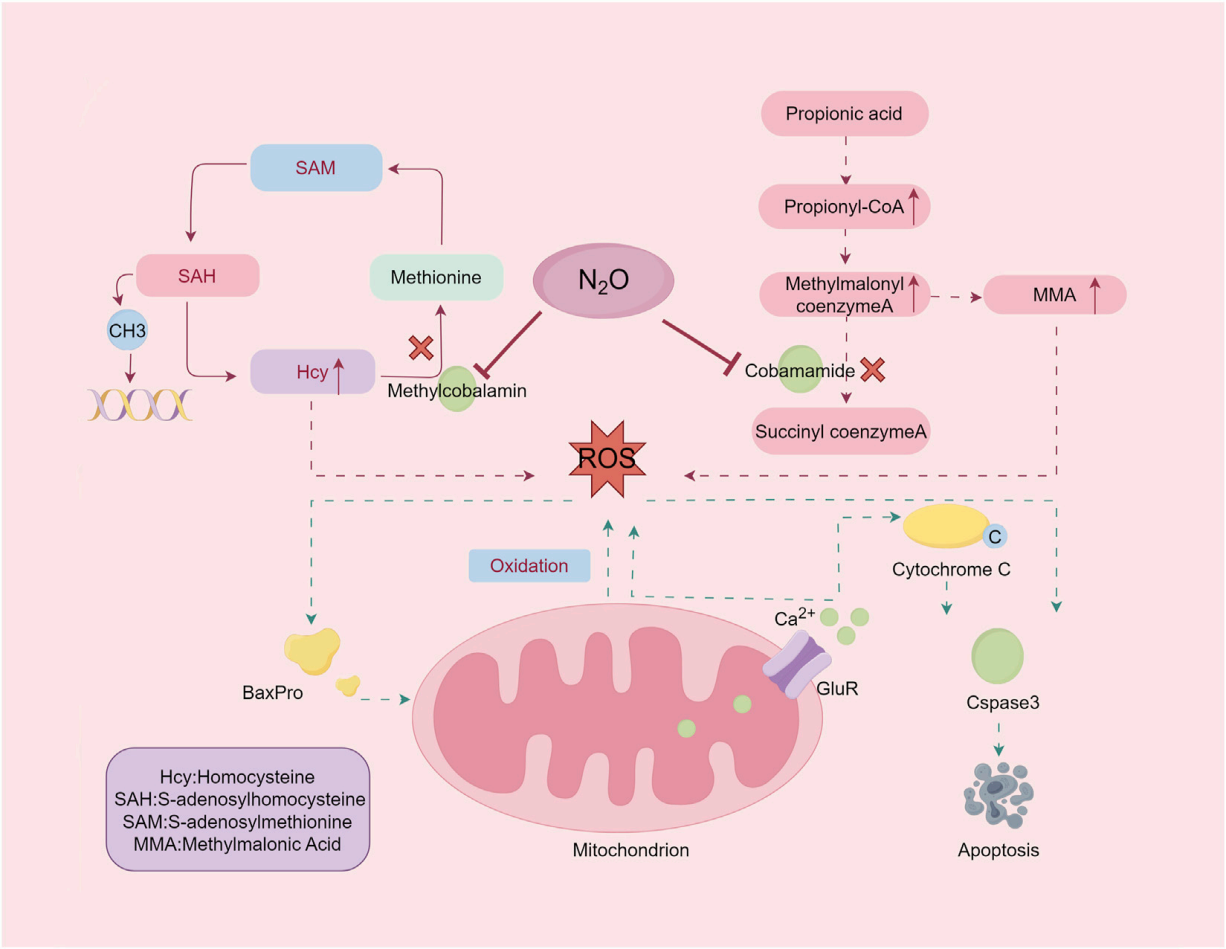
The damage mechanism of nitrous oxide. Exposure to nitrous oxide leads to inactivation of adenosine cobalamin and methylcobalamin, resulting in elevated levels of the substrates MMA and Hcy. Excessive accumulation of MMA and homocysteine induces oxidative stress. By Figdraw.

Vit B12 is a cobalt-containing molecule essential for various biochemical reactions, primarily in its active forms adenosylcobalamin and methylcobalamin (Froese et al., 2019). These forms are critical in the pathogenesis of N₂O poisoning. N₂O exposure oxidizes the cobalt ion in Vit B12 from its +1 to +3 oxidation state, irreversibly inactivating the molecule (Garakani et al., 2016). This inactivation disrupts key enzymatic reactions, including the conversion of methylmalonyl-CoA to succinyl-CoA by methylmalonyl-CoA mutase and the synthesis of methionine from homocysteine (Hcy) by methionine synthase (Figure 2). The downstream biochemical consequences of this disruption form the basis for understanding the neural damage and clinical manifestations induced by N_2_O.

The accumulation of methylmalonic acid (MMA) and Hcy because of these disruptions leads to oxidative damage and impaired methylation. Methionine, which is converted into S-adenosylmethionine (SAM), is crucial for methylation processes affecting DNA, RNA, proteins, and lipids. Disruption of SAM synthesis affects cellular integrity and function. Understanding these mechanisms elucidates the biochemical basis of N₂O-induced neurotoxicity and its clinical manifestations.

### 6.2 Neural damage mechanisms

The molecular disruptions outlined above initiate a series of cascading effects, leading to neural damage through oxidative stress, excitotoxicity, and demyelination. The following sections detail the interconnected mechanisms underlying N₂O-induced neurotoxicity.

#### 6.2.1 Oxidative damage

The accumulation of Hcy and MMA leads to oxidative stress, a critical factor in N₂O-induced neurotoxicity. Hcy self-oxidation generates ROS, disrupts redox homeostasis, and induces neuronal apoptosis via upregulation of Bax protein expression and activation of the caspase-3 pathway (Hu et al., 2019). Excessive activation of the ionotropic and metabotropic glutamate receptors by Hcy exacerbates excitotoxicity, leading to intracellular calcium overload, mitochondrial dysfunction, and apoptosis. This oxidative damage links molecular disruptions to more extensive neural damage, emphasizing the interplay between biochemical alterations and neuronal apoptosis.

MMA further contributes to oxidative stress by impairing the mitochondrial respiratory chain complexes and energy metabolism. This cascade of oxidative damage to proteins, lipids, and DNA highlights the interconnected pathways through which N₂O affects neural integrity (Guo et al., 2024).

#### 6.2.2 Excitotoxicity

N₂O acts as a noncompetitive inhibitor of NMDAR, disrupting their regulatory role in synaptic plasticity, memory, and learning (Bae et al., 2022). The persistent activation of NMDAR by Hcy and MMA leads to prolonged excitotoxicity, impairs hippocampal function, and contributes to cognitive deficits (Poddar and Paul, 2009). Additionally, N₂O-induced dopamine dysregulation within the limbic system may underlie psychiatric symptoms, such as euphoria, agitation, and hallucinations (Wu et al., 2021). Together, oxidative stress and excitotoxicity form a dual mechanism that underpins the neural damage caused by N_2_O exposure.

#### 6.2.3 Demyelinating injury

##### 6.2.3.1 Myelination disorder

Human myelination begins in mid-gestation and peaks during the first few years after birth, continuing into adulthood (Purger et al., 2016). Myelin, which is synthesized by oligodendrocytes, relies on MBPs for structural stability and function. SAM-dependent methylation is critical for MBP function (Friess et al., 2016). N₂O exposure disrupts methionine and SAM synthesis, impairing MBP methylation and leading to demyelination. Studies have demonstrated that methionine supplementation partially mitigates these effects (van der Westhuyzen et al., 1982). This demyelination is both a consequence of N_2_O-induced biochemical disruptions and a key factor in the clinical manifestations of its neurotoxicity.

##### 6.2.3.2 Myelin vacuolization and demyelination

N₂O-induced Vit B12 inactivation results in MMA accumulation (Hathout and El-Saden, 2011), altering lipid composition in myelin membranes and destabilizing their structure (Agamanolis et al., 1978). Histopathological evidence has linked vacuolated myelin and demyelination to abnormalities in the molecular structure of MBP (Weil et al., 2016). These findings underscore the critical role of methylcobalamin in the maintenance of neural integrity.

##### 6.2.3.3 Cytokine dysregulation and myelin swelling

Elevated levels of proinflammatory cytokines, such as TNF-α, and reduced levels of epidermal growth factor have been observed in Vit B12-deficient states (Buccellato et al., 1999). This imbalance contributes to myelin swelling and axonal damage (Scalabrino et al., 2000). Therapeutic strategies targeting cytokine regulation hold potential for mitigating N₂O-related neuropathology.

### 6.3 Clinical manifestations of nervous system injury

N₂O-induced injuries are classified into acute, subacute, and chronic phases. Acute manifestations include hypoxia, frostbite, and psychiatric symptoms. Subacute and chronic injuries predominantly feature demyelination of the nervous system and megaloblastic anemia, with up to 96% of patients experiencing neurological damage (Oussalah et al., 2019; Dawudi et al., 2024).

Spinal Cord Lesions: Symptoms include gait instability, sensory deficits, and motor weakness, often progressing to paraplegia in severe cases (Choi et al., 2019; Mair et al., 2023). Peripheral neuropathy manifests as numbness, tingling, and limb weakness, predominantly affecting the lower extremities (Winstock and Ferris, 2020). Cognitive and psychiatric symptoms include memory impairment, disorientation, and psychiatric conditions, such as paranoia and agitation.

### 6.4 Auxiliary examinations

#### 6.4.1 Imaging characteristics

MRI findings often reveal T2 hyperintensities in the posterior columns of the spinal cord and subcortical white matter (Su et al., 2000; Graber et al., 2010; Assaf et al., 2020). These imaging features overlap with those observed in Vit B12 deficiency, emphasizing the importance of differential diagnosis.

#### 6.4.2 Laboratory and neurophysiological examinations

Decreased Vit B12 levels and elevated Hcy and MMA are hallmark findings in N₂O-induced neuropathy (Warendorf et al., 2022). Neurophysiological studies frequently reveal mixed axonal and demyelinating neuropathies (Li et al., 2016).

### 6.5 Diagnosis and differential diagnosis

Based on clinical experience, the diagnostic criteria for neurological damage due to long-term N₂O inhalation include the following: ① A history of N₂O exposure, particularly frequent short-term inhalations. ② Subacute or chronic progression of symptoms indicating neurological impairment. ③ MRI and electrophysiological evidence of damage to the spinal cord and/or peripheral nerves. ④ Exclusion of other potential causes, such as immune, metabolic, infectious, toxic, and neoplastic conditions, via auxiliary tests. When young patients exhibit symptoms, such as deep or superficial sensory disturbances, motor dysfunction, cognitive deficits, or psychiatric issues, further investigation is warranted. Notably, if these symptoms coincide with an inverted “V”-shaped T2 hyperintensity on spinal MRI, elevated serum Hcy and MMA levels, and normal or reduced Vit B12 levels, it is crucial to inquire about past N₂O inhalation. A confirmed history of N₂O use, along with the exclusion of other causes, supports the diagnosis.

For patients exhibiting neurological symptoms, it is crucial to differentiate between various diseases that may present similarly, such as Guillain-Barré syndrome, multiple sclerosis, and copper deficiency myelopathy. Accurate diagnosis is essential for effective treatment and management of these conditions.

Guillain-Barré syndrome typically follows a preceding infection and manifests as an acute onset of symptoms that progressively worsen, usually peaking around 2 weeks. Cerebrospinal fluid examination in such cases may reveal protein-cell dissociation. Multiple sclerosis predominantly affects women and is characterized by a relapsing course. Brain MRI often shows the characteristic Dawson’s fingers, whereas spinal cord lesions usually extend to fewer than two vertebral segments. In multiple sclerosis, cerebrospinal fluid often contains oligoclonal IgG bands. Copper is a vital cofactor for methionine synthase, and its deficiency can impair the enzyme’s function, disrupting methylation reactions. This disruption leads to neurological and hematological damage similar to that seen in N_2_O abuse, including posterior column lesions in the cervical and thoracic spinal cord, demyelination, or axonal injury of peripheral nerves. These manifestations are challenging to differentiate clinically and radiologically. Copper deficiency myelopathy is more likely to occur in patients who have undergone upper gastrointestinal surgery, typically between the ages of 50 and 60 years, and is more common in women than in men. Biochemical tests in these patients may reveal decreased serum copper and ceruloplasmin levels (Kumar et al., 2004; Jaiser and Winston, 2010) (Table 2).

**TABLE 2:** Differential diagnosis of diseases with clinical manifestations similar to neurological damage caused by N_2_O abuse.

	Neurological damage caused by N_2_O abuse	Guillain-Barré Syndrome	Multiple clerosis	Copper eficiency Myelopathy	References
Age/Gender	Predominantly younger population	Any age group	Onset in early adulthood, with females being more affected than males	Most common in the 50–60 age group, with females more affected than males	Jaiser and Winston (2010)
Lesion site	Central and peripheral nervous systems	Peripheral neuropathy	Central nervous system	Central and peripheral nervous systems	-
Characteristics of spinal cord MRI	High T2 signal in the posterior and lateral columns of the spinal cord	-	Lesion length is less than two vertebral segments, distributed in the white matter	T2 hyperintensity in the dorsal column of the spinal cord	-
Affected segments of the spinal cord	More commonly seen in the cervical spinal cord, followed by the thoracic spinal cord	-	Commonly observed in the cervical and thoracic sections	Commonly observed in the cervical and thoracic sections	-
Serological characteristics	Reduction in Vit B12, increase in Hcy and MMA	Positive for ganglioside antibodies	-	Serum copper, ceruloplasmin levels decreased	Kumar et al. (2004)
Specific markers/antibodies	-	Cerebrospinal fluid protein-cell dissociation phenomenon	Intrathecal synthesis of IgG, presence of IgG oligoclonal bands in cerebrospinal fluid, none in serum	-	-
Brain involvement	No reports found	-	Periventricular areas, corpus callosum, brainstem, and cerebellum	No reports found	-
Peripheral nerve involvement	Demyelination and axonal damage, more severe in the lower limbs	Sensory, motor, and autonomic nerves, demyelination or axonal pathology	-	Axonal damage, demyelination	-
Treatment	Supplementation with Vit B12, B6, folic acid, and methionine	Human immunoglobulin therapy, plasmapheresis	Immunosuppressive and modulatory therapy	Copper supplementation	-
Prognosis	Most respond well to treatment, but some patients sustain permanent damage	Unidirectional disease course, peak condition reached within approximately 2 weeks, recovery within several weeks to months	The majority have a good prognosis, while those with older onset age, or with extrapyramidal or cerebellar dysfunction, tend to have a worse prognosis	Timely supplementation of copper can prevent disease progression, with only a few patients experiencing partial relief	-

### 6.6 Treatment strategies

Immediate cessation of N₂O exposure is critical. Vit B12 replacement therapy, typically administered intramuscularly, is the cornerstone of treatment (Marotta and Kesserwani, 2020). Adjunctive supplementation with folic acid and vitamin B6 enhances recovery by supporting the methylation cycles and reducing neurotoxicity (Algahtani et al., 2020). Early intervention improves the prognosis, although severe axonal damage may result in permanent deficits.

## 7 Heroin

**FIGURE 3:**
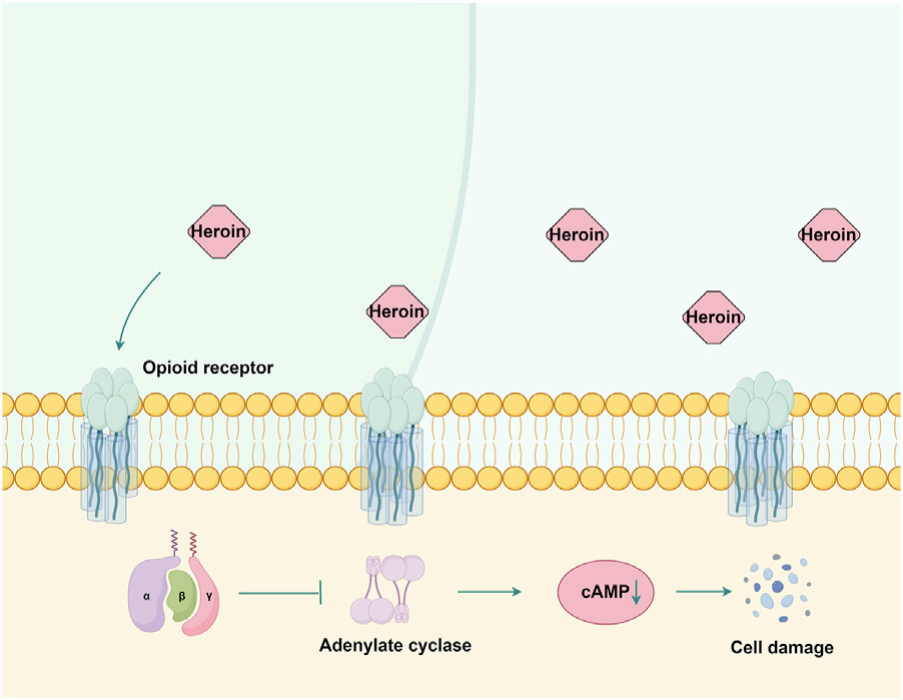
The mechanism of action of opioid receptors in heroin. Heroin primarily exerts its effects by binding to μ, δ, and κ opioid receptors. This interaction inhibits adenylate cyclase activity and reduces cAMP production, subsequently causing damage to nerve cells. By Figdraw.

Heroin, chemically known as 3,6-diacetylmorphine, is a highly addictive opioid that binds to μ, δ, and κ opioid receptors (Nobay et al., 2004). These G protein-coupled receptors (Waldhoer et al., 2004) undergo conformational changes upon heroin binding, thereby inhibiting adenylate cyclase activity, reducing cAMP production (Tedford and Zamponi, 2006), and subsequently impairing neuronal signaling (Figure 3). Prolonged heroin use leads to receptor downregulation and desensitization mediated by G protein-coupled receptor kinase and arrestin (Pasternak, 2012). The effects of heroin on GABAergic interneurons in the ventral tegmental area enhance dopamine release in the nucleus accumbens (Kim et al., 2015), producing euphoria (Saurer et al., 2009).

### 7.1 Neurotoxicity and cognitive impairments

Heroin-induced oxidative stress disrupts the balance between oxidation and antioxidation (Xu et al., 2006), leading to neuronal death and BBB disruption during withdrawal (Sharma and Ali, 2006). Studies have indicated that heroin reduces mitochondrial potential and alters the Bcl-2/Bax ratio, thereby affecting cell survival (Cunha-Oliveira et al., 2007). Chronic use activates microglial cells in the cortical gray matter, thereby promoting inflammation and neurotoxicity. Heroin-related brain hypoxia results primarily from respiratory depression, which exacerbates ischemic risk (Solis et al., 2017).

### 7.2 Neurological damage and psychiatric symptoms

Heroin injection significantly increases the risk of cerebrovascular complications, including ischemic and hemorrhagic stroke (Sloan et al., 1991). Inhalation methods, such as “chasing the dragon,” are associated with leukoencephalopathy, characterized by progressive motor and cognitive impairments, ataxia, and spastic paresis (Keogh et al., 2003). MRI revealed symmetrical white matter lesions in the corticospinal tract (Wolters et al., 1982), while diffusion tensor imaging highlighted impaired white matter connectivity due to myelin damage rather than axonal injury (Bora et al., 2012).

Heroin also induces psychiatric symptoms, such as depression, anxiety, impulsiveness, and interpersonal sensitivity (Pasternak, 2012). Cognitive impairments, including reduced memory, impaired impulse control, and decision-making difficulties (Tolomeo et al., 2021), are commonly observed.

### 7.3 Treatment strategies

Calcium channel blockers and opioid antagonists, such as naltrexone, have shown potential for managing heroin addiction and ischemic stroke (Peyravian et al., 2022). Emerging therapies, including nanotechnology-based drug delivery, are promising for reversing long-term neuronal damage (Nguyen et al., 2021).

### 7.4 Comparative analysis: cocaine and heroin

Although cocaine and heroin have profound neurobiological and vascular effects, their mechanisms of action differ significantly. Cocaine primarily acts as a psychostimulant by increasing monoamine neurotransmitter levels, leading to excitotoxicity and vascular damage. Heroin, an opioid, primarily modulates G protein-coupled receptor activity and affects neuronal excitability and reward pathways. Despite these differences, both substances contribute to oxidative stress, neuroinflammation, and cognitive impairment, highlighting the overlapping pathological pathways.

The vascular effects of cocaine include angiogenesis and vasoconstriction, in contrast to heroin’s predisposition to ischemic and hemorrhagic complications. Both substances are linked to leukoencephalopathy; however, heroin-induced leukoencephalopathy lacks the spongiform quality characteristic of prion disease. Understanding these similarities and differences is critical for developing targeted interventions for substance abuse.

## 8 Conclusion

This review highlights the neurotoxic effects of six recreational drugs, namely, METH, cocaine, synthetic cathinones, ketamine, nitrous oxide, and heroin. Despite their diverse pharmacological properties, these substances converge on shared mechanisms of neurotoxicity, including oxidative stress, mitochondrial dysfunction, excitotoxicity, and neuroinflammation. The resulting neuronal damage manifests as a spectrum of cognitive, psychiatric, and motor dysfunctions, with long-term consequences for brain structure and function. Psychostimulants (METH, cocaine and synthetic cathinones) induce neurotoxicity primarily through disruption of monoaminergic signaling. Chronic use of these substances is strongly associated with persistent cognitive deficits, psychiatric symptoms, and, in some cases, neurovascular damage. Dissociative anesthetics (ketamine and nitrous oxide) impair glutamatergic neurotransmission and mitochondrial function, contributing to excitotoxicity and neurodegeneration. Their neurotoxic effects are dose- and frequency-dependent, with chronic use exacerbating neuronal apoptosis and neuroinflammation. Opioids (heroin) predominantly target the brain’s reward system, leading to microglial activation and oxidative stress. Chronic heroin use is linked to ischemic and hemorrhagic strokes, as well as diffuse leukoencephalopathy.

Although significant progress has been made in elucidating the molecular and cellular mechanisms underlying drug-induced neurotoxicity, key questions remain. For example, the roles of genetic predisposition, age, and comorbidities in modulating susceptibility to neurotoxicity remain underexplored. Additionally, the bidirectional relation between neuroinflammation and neuronal injury requires further investigation.

Future studies should prioritize examining recreational drugs, such as MDMA and cannabis, which have been widely consumed for decades and continue to pose significant public health challenges. Cannabis, in particular, is one of the oldest recreational substances with a long history of use, while MDMA has been extensively studied for its recreational effects since its emergence several decades ago. These substances are not included in the present review due to space constraints; however, readers are encouraged to explore the extensive body of research focusing on their neurological impacts for a broader understanding. In parallel, therapeutic strategies should focus on targeting the shared mechanisms of neurotoxicity, such as oxidative stress and neuroinflammation, while considering drug-specific interventions. For instance, NMDAR antagonists and antioxidants show promise in mitigating the damage caused by dissociative anesthetics, whereas dopamine reuptake inhibitors and CBT may be more effective for psychostimulant-induced impairments. Additionally, longitudinal studies are crucial for evaluating the efficacy and safety of these treatments, particularly in vulnerable populations, such as adolescents and individuals with pre-existing conditions.

By addressing these knowledge gaps, researchers and clinicians can develop more effective prevention and intervention strategies to mitigate the long-term neurological and societal effects of recreational drug use.
